# Four decades of measuring stillbirths and neonatal deaths in Demographic and Health Surveys: historical review

**DOI:** 10.1186/s12963-020-00225-0

**Published:** 2021-02-08

**Authors:** Joseph Akuze, Simon Cousens, Joy E. Lawn, Peter Waiswa, Vladimir Sergeevich Gordeev, Fred Arnold, Trevor Croft, Angela Baschieri, Hannah Blencowe

**Affiliations:** 1 Maternal, Adolescent, Reproductive & Child Health (MARCH) Centre, London School of Hygiene & Tropical Medicine, London, UK; 2 Centre of Excellence for Maternal Newborn and Child Health Research, Dept. of Health Policy, Planning and Management, Makerere University School of Public Health, Kampala, Uganda; 3 Global Health Division, Karolinska Institutet, Stockholm, Sweden; 4 Institute of Population Health Sciences, Queen Mary University of London, London, UK; 5 The Demographic and Health Surveys (DHS) Program, ICF, Rockville, USA

**Keywords:** Stillbirths, Neonatal deaths, Demographic and Health Surveys, Questionnaires

## Abstract

**Background:**

Worldwide, an estimated 5.1 million stillbirths and neonatal deaths occur annually, 98% in low- and middle-income countries. Limited coverage of civil and vital registration systems necessitates reliance on women’s retrospective reporting in household surveys for data on these deaths. The predominant platform, Demographic and Health Surveys (DHS), has evolved over the last 35 years and differs by country, yet no previous study has described these differences and the effects of these changes on stillbirth and neonatal death measurement.

**Methods:**

We undertook a review of DHS model questionnaires, protocols and methodological reports from DHS-I to DHS-VII, focusing on the collection of information on stillbirth and neonatal deaths describing differences in approaches, questionnaires and geographic reach up to December 9, 2019. We analysed the resultant data, applied previously used data quality criteria including ratios of stillbirth rate (SBR) to neonatal mortality rate (NMR) and early NMR (ENMR) to NMR, comparing by country, over time and by DHS module.

**Results:**

DHS has conducted >320 surveys in 90 countries since 1984. Two types of maternity history have been used: full birth history (FBH) and full pregnancy history (FPH). A FBH collecting information only on live births has been included in all model questionnaires to date, with data on stillbirths collected through a reproductive calendar (DHS II-VI) or using additional questions on non-live births (DHS-VII). FPH collecting information on all pregnancies including live births, miscarriages, abortions and stillbirths has been used in 17 countries. We found no evidence of variation in stillbirth data quality assessed by SBR:NMR over time for FBH surveys with reproductive calendar, some variation for surveys with FBH in DHS-VII and most variation among the surveys conducted with a FPH. ENMR:NMR ratio increased over time, which may reflect changes in data quality or real epidemiological change.

**Conclusion:**

DHS remains the major data source for pregnancy outcomes worldwide. Although the DHS model questionnaire has evolved over the last three and half decades, more robust evidence is required concerning optimal methods to obtain accurate data on stillbirths and neonatal deaths through household surveys and also to develop and test standardised data quality criteria.

## Key findings

**Table:** 

**What is new?**	
**• What was known already**: Demographic and Health Surveys (DHS) have been the main source of information on child mortality in most low- and middle-income countries over the past three and a half decades, and the major data input for two thirds of the world’s estimated 5.1 million stillbirths and neonatal deaths. **• What was done:** Survey tools have evolved over time, but these changes and the potential effects on national stillbirth and neonatal mortality data have not been systematically assessed before. Our study addresses this gap.	
**What changed in DHS over time?**	
**•** From 1984 to date, the DHS programme had seven phases (DHS-I to DHS-VII) collecting data from more than 400 surveys in more than 90 countries. The model questionnaires are revised for each phase with two main approaches for capturing information on births: ◦ Full birth history (FBH), capturing a woman’s lifetime live births and survival status, is used to calculate neonatal and child mortality. Throughout all DHS phases, the model questionnaire included an FBH and most countries have implemented this approach. Minor changes to the FBH have been made during the last three decades, including adding and then refining a question to capture omitted child deaths (DHS-III to DHS-V) and introducing a question of the day of death in DHS-VII. ◦ Full pregnancy histories (FPH) capture miscarriages, terminations of pregnancy and stillbirths, as well as live births. FPH has been used by DHS in 17 countries (five in Central Asia, two in Southeast Asia, two in Western Asia, two in Africa, two in Eastern Europe and one in Latin America). **•** Stillbirths were initially not captured or reported in DHS-I. In DHS-II to DHS VI, reproductive calendars were used to generate stillbirth data. Since DHS-III, stillbirth data have been shown in the standard national DHS tabulation. DHS-VII introduced a reverse truncated history for non-live births in the last 5 years.	
**What changed in the data over time?**	
**• DHS data quality assessment criteria**: Neonatal deaths in the DHS programme include sex ratios at birth and of neonatal deaths; heaping of neonatal deaths on day 7; and the proportion of infant deaths that are in the neonatal period. These are all problematic as may be due to true epidemiological change, not just data quality. **• Data quality for stillbirths**: Data quality for stillbirths is often assessed by SBR:NMR ratio. Our assessment of SBR:NMR suggested that the ratio did not change across DHS-II to DHS-VI, and stillbirth data seem mostly low quality for surveys conducted in DHS-VII. Using FPH, stillbirth data quality are more variable, with some apparently higher quality, which may be related to the use of differing pregnancy history tools and varied implementation between surveys. Contextual societal barriers to reporting pregnancy loss may also play a role.	
**What next in measurement and research?**	
**• Measurement improvement now:** From 2020, the DHS programme (DHS-VIII) has changed its model questionnaire to be based on FPH. This change was influenced by the EN-INDEPTH study’s randomised comparison of the two approaches, showing higher reporting of stillbirths but not neonatal deaths with FPH, compared with FBH. However, whilst FPH may improve capture of stillbirths, optimising data quality is also dependent on survey implementation including training and supervision of data collectors, optimal use of electronic platforms, plus addressing contextual barriers to women reporting pregnancy losses. **• Research needed:** More research is required to develop robust measures of data quality for stillbirths and neonatal deaths.	

## Background

There were an estimated 5.1 million stillbirths and neonatal deaths worldwide in 2018. 98% of these deaths occurred in low- and middle-income countries (LMICs), with over 75% in sub-Saharan Africa and South Asia [1–5]. These deaths have an impact on women, families, health-workers and wider society [6], yet the majority are preventable through high quality antenatal, childbirth and newborn care [1, 7, 8]. Measuring and monitoring trends in stillbirth and neonatal mortality, therefore, provides an important indicator of maternal health and access to high-quality care [9, 10].

However, whilst high-income countries have national civil and vital registration statistics (CRVS) systems that record these outcomes in a timely and reliable way, CRVS systems in most LMICs are limited in coverage and quality. Even when such CRVS systems capture adult outcomes, there is known to be selective under-reporting of neonatal deaths (especially preterm neonates) and even more so of stillbirths [11]. At the global level, fewer than 5% of all stillbirths and neonatal deaths are captured in CRVS; this is not much higher for under-5 child deaths [2]. LMICs therefore rely on population-level household surveys for data on these indicators [12]. Indeed, as LMICs account for the majority of the world’s births and an even higher proportion of child deaths and stillbirths, such survey data are the main input for estimating over two thirds of the burden worldwide.

The largest survey platforms used for estimating child mortality include Demographic Health Surveys (DHS), Reproductive Health Surveys (RHS), Pan Arab Project for Family Health (PAPFAM) surveys and UNICEF’s Multiple Indicator Cluster Surveys (MICS). However, PAPFAM and most MICS do not include stillbirths, and RHS are mainly conducted in middle-income countries. Only DHS has systematically captured stillbirths and neonatal deaths in LMICs throughout most of its history, and hence, this paper focuses on the approaches taken over time by DHS to capture these outcomes.

**Fig. 1:**
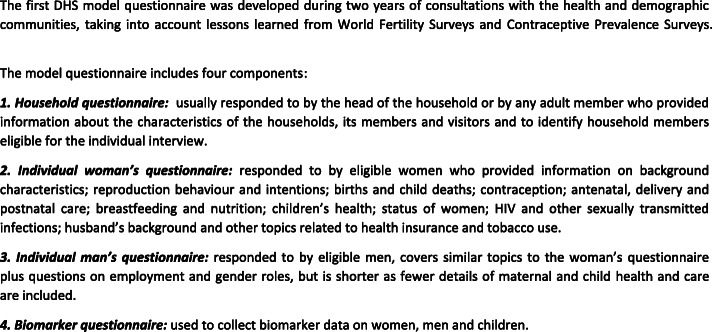
DHS model questionnaire overview of content

The DHS programme, primarily funded by the United States Agency for International Development (USAID), is a follow-on to the World Fertility Surveys and Contraceptive Prevalence Surveys that were conducted between 1972 and 1984 to collect data on fertility, mortality and contraceptive use [13–15]. A large focus of the DHS programme remained on analysis of fertility patterns and trends, and child mortality; however, in addition, it also collects information on maternal and child health, nutrition, human immunodeficiency virus (HIV) and acquired immunodeficiency syndrome (AIDS), malaria, domestic violence and other country-specific indicators of interest [13, 16]. The DHS programme uses a basic approach of collecting comparable data across countries using a model questionnaire which is revised every 5 years through a consultation process (see Fig. 1) [17]. DHS is currently in the 8th phase of its programme [15].

Omission of stillbirths and neonatal deaths in surveys is known to affect the data quality of these indicators; however, assessing data quality for stillbirths and neonatal deaths in surveys is challenging in the absence of high-quality population-based data with which to compare survey estimates. The ratio of stillbirths to overall neonatal mortality rates (SBR:NMR), which detects where stillbirths are under-reported compared with neonatal deaths, has been used as a stillbirth data quality criterion [18, 19]. High-quality historical data from high-income countries report ratios of at least 1 for countries with NMRs of 10–35 per 1000, and similar ratios may be expected from LMICs [20, 21]. DHS data quality assessments for neonatal deaths have used several criteria including sex ratios at birth and of neonatal deaths and heaping of neonatal deaths on day 7 and the proportion of infant deaths that are in the neonatal period [22]. However, the latter has limited utility due to well-documented epidemiological variations with ratios varying by mortality contexts [23, 24]. As early (days 0–6) neonatal deaths are the most frequently omitted deaths, the proportion of neonatal deaths that occurred on days 0–6 (or the early to overall neonatal mortality (ENMR:NMR) ratio) is another potential marker of data quality [25].

Challenges to collecting data on stillbirths and neonatal deaths in surveys have led to a variety of approaches being used over time. No previous studies have systematically described these, how they have evolved over time or the effect of these changes on indicator comparability over time. The objectives of this paper are for DHS phases I–VII are as follows:

1. ***Provide an overview of the measurement of stillbirths and neonatal deaths*** and how this has changed over time.
2. ***Review DHS data on stillbirths and neonatal deaths, and their performance*** against potential markers of data quality.

## Methods

We conducted a review between the 17th of November 2017 and 9th of December 2019. We searched POPLINE and PubMed databases and the DHS website using combinations of key words including “*Birth History”, “Pregnancy History”, “Questionnaire”, “World Fertility Surveys”, “Demographic and Health Surveys”, “stillbirth”*, “perinatal death”, “neonatal death”, “child death”, “perinatal mortality”, “neonatal mortality” and “child mortality” for reports and journal articles published since 1982 with a focus on the implementation of DHS for capturing stillbirths and neonatal deaths.

We obtained the DHS’s model woman’s questionnaires for all DHS phases from the DHS website and reviewed all the eleven previous model questionnaires (DHS model questionnaires: I-A, I-B, II-A, II-B, III-A, III-B, IV-A, IV-B, V, VI and VII), reports and journal articles for information relevant to survey implementation relating to the measurement of stillbirths and neonatal deaths in the questionnaire’s reproduction section.

We extracted summary data from the DHS website’s STATcompiler on stillbirths and neonatal deaths from all surveys from 1984 to December 2019. The extracted data were exported first to Microsoft Excel spreadsheets and then imported into Stata 16.0 for further data management and analysis. These data were analysed using descriptive and geospatial techniques using choropleth maps in Stata version 16.0.

We summarised data quality for neonatal mortality and stillbirth rate data over time and by data collection method using a single available measure for each outcome focused on the detection of omission: SBR:NMR ratio for stillbirths and ENMR:NMR ratio for neonatal mortality. Results are presented using descriptive statistics and graphical summaries such as two-way scatter plots. We compared mean SBR, mean NMR, SBR:NMR and ENMR:NMR ratios by DHS phase and type of module implemented using statistical tests for trend and differences in proportions, and for SBR:NMR using box plots.

## Results

### Overview of the measurement of stillbirths and neonatal deaths in DHS

The predominant method to collect information on neonatal deaths (deaths in the first 28 days of life) in DHS has been through the use of full maternity histories. To collect information on stillbirths, both full maternity histories and reproductive calendars have been used. As neither of these approaches allows capture of pregnancy length in weeks or days, it is not possible to apply standard ICD-11 stillbirth definitions, and a pregnancy loss at seven or more months of gestation is used to approximate late fetal deaths or stillbirths [26].

### Maternity history approach

In the DHS, full maternity histories were introduced more than three decades ago to gather retrospective data on women’s fertility, births and infant and child deaths [12]. This is in contrast to many other surveys which used summary birth histories, collecting information only on the number of children ever born and the number that survived, and then using indirect methods to estimate overall child mortality rates only [27–29]. From the mortality estimation perspective, these questions were initially predominantly used to estimate infant and overall under-5 mortality rates; however, as information on the precise age at death was included in the full maternity history, it is possible to also estimate neonatal mortality from these questions.

Two types of full maternity histories have been implemented in DHS: full birth history (FBH) and full pregnancy history (FPH) [30]. Both the FBH and FPH are administered in the woman’s questionnaire to women aged 15–49 years who consent to participate in the survey. These modules are implemented in the reproduction section of the woman’s questionnaire (Additional file 1). The FBH collects information on all pregnancies that resulted in a live birth, survival status of the child and where relevant the age at death. Data on stillbirths are not collected directly in an FBH. The FPH collects information on all pregnancy outcomes including miscarriages, terminations of pregnancy, stillbirths and live births. As with the FBH, the survival status of all live births and where relevant the age at death is included [30].

Both full maternity histories have predominantly been implemented in DHS using a forward approach, starting with the earliest events and detailing each birth or pregnancy in time order [12, 30]. Whilst there is some evidence from the World Fertility Survey that a backward approach starting with the latest event is associated with more detailed probing of later events and slightly fewer missed or time-displaced events, the advantages were not considered sufficient to change the standard DHS approach [12, 31, 32].

To seek to reduce the length of the survey tool, early surveys in Peru and the Dominican Republic experimented with using a back truncated approach, collecting data on events only in the last 5 or 6 years [33, 34]. Overall, they found similar data quality compared with the full maternity history approach. Whilst there was some improvement in the quality of reporting of dates of very recent events, in Peru displacement of events prior to the 5-year period was found, and therefore, this approach has not been adopted in standard DHS.

### Reproductive calendar approach

Reproductive calendars collect information on pregnancies, births and contraceptive use by month for the 5-year period preceding the survey. The use of a reproductive calendar first developed in experimental studies in Peru and Dominican Republic in 1986 was found to improve the accuracy of the capture of contraceptive use within surveys [33, 35, 36]. As reproductive calendars record for each month of the preceding 5 years whether a woman was using contraception, was pregnant or gave birth/had a pregnancy end, these data can be used to estimate stillbirth rates [37].

### Evolution of DHS’s FBH reproduction section in the woman’s questionnaire for **p**hases I to VII

**Fig. 2:**
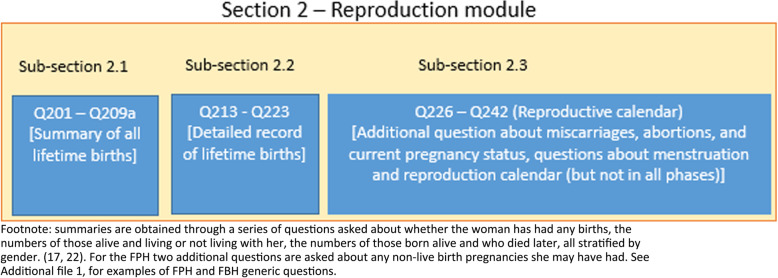
Overview of reproduction section used in Demographic and Health Surveys (DHS) questionnaire

**Fig. 3:**
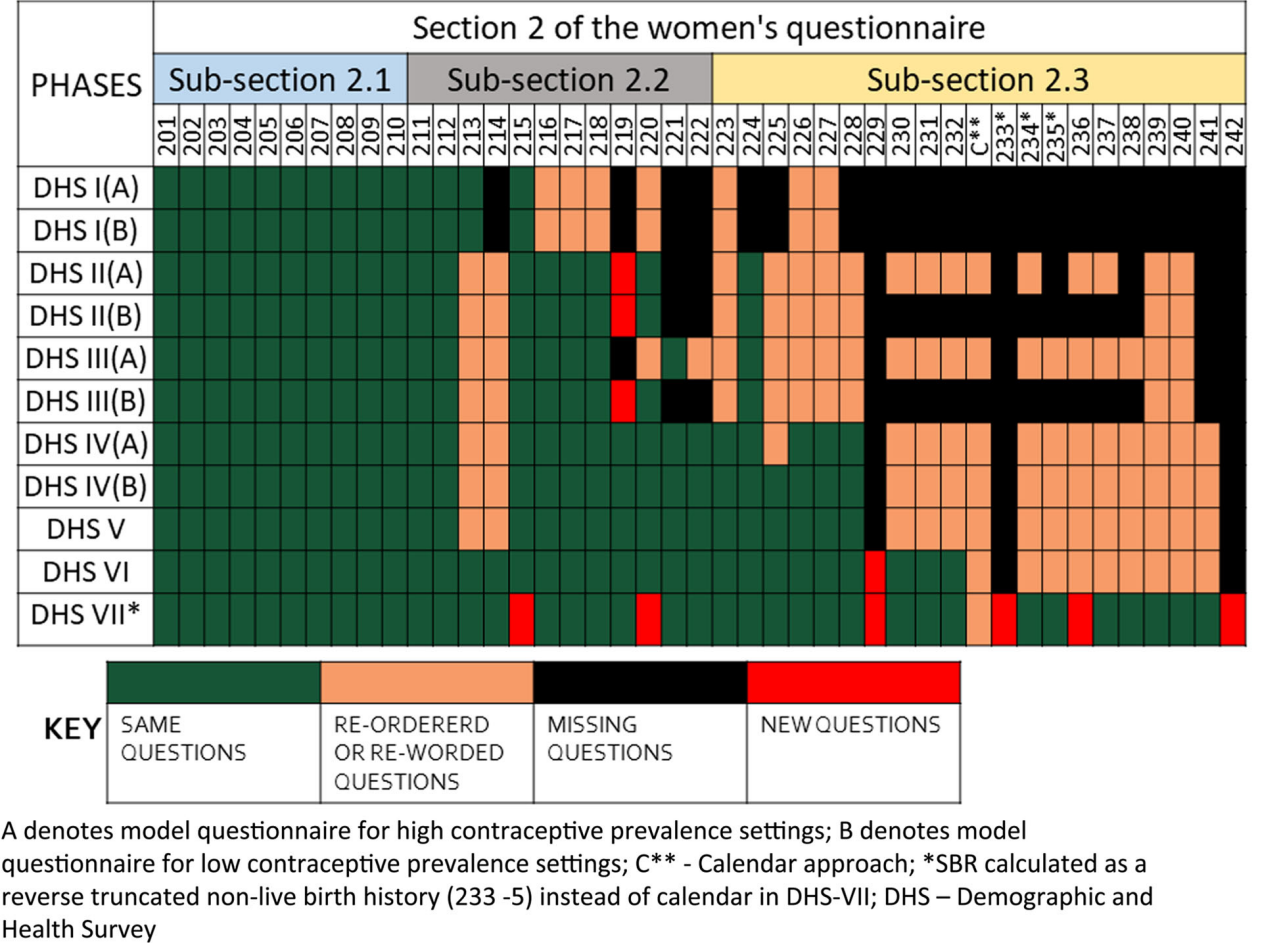
Comparing the model Demographic and Health Surveys’ full birth history (FBH) questionnaire across phases I–VII

The DHS’s woman’s questionnaire contains a reproduction section which comprises three sub-sections (Fig. 2). This section has evolved through the DHS phases with additions of new questions and removal or modifications of questions. In phases I to IV, two separate model woman’s questionnaires were in use— Questionnaire A for high contraceptive prevalence countries and Questionnaire B for countries with low prevalence of contraceptive use. Sub-section 1 did not change across phases. More changes that are substantial were made to sub-sections 2.2 and 2.3, particularly in later phases (Fig. 3 and Additional file 2).

Methods to estimate child mortality, including neonatal mortality, have not changed substantially throughout the DHS phases and do not vary between FBH and FPH. Questions around the precise age at death for any deceased children have been included in all DHS phases. These include information in days if less than 1 month, in months if less than 2 years, otherwise in years. In DHS-III, an interviewer calculation and probe where added to investigate the potential omission of children who had died in all cases with a reported birth interval of more than 4 years. In DHS-IV onwards, this was simplified to probe simply if there were any other live births between each reported birth; from DHS-V onwards, this was expanded to specify “*Were there any other live births between (NAME OF PREVIOUS LIVEBIRTH) and (NAME) including any children who died after birth*?” Reported child mortality has been disaggregated to show neonatal mortality rates in standard DHS reports since DHS-II.

There has been much greater variation in the collection of data to inform estimates of stillbirths in DHS over time. A FBH alone does not capture any information on non-live births. No questions enabling stillbirth rate estimates were included in the model woman’s questionnaire prior to 1993 (DHS-I). In DHS-II, a reproductive calendar to capture pregnancy, birth and contraceptive use was included in the model woman’s questionnaire A for high contraceptive prevalence countries and in DHS-IV the reproductive calendar was also added to the model woman’s questionnaire B for low contraceptive prevalence countries. However, in both cases, the reason stated for including these questions was to avoid misclassifying months in which the woman is pregnant as months of exposure to the risk of pregnancy when calculating rates of contraceptive failure and discontinuation rather than to be able to estimate perinatal mortality. Therefore, despite this information being collected, estimates of stillbirth rates were infrequently included in the final DHS reports for countries using the standard approach of FBH and a reproductive calendar. Hence, whilst stillbirth rates can be calculated retrospectively from these surveys, and are included in the DHS platform’s STATcompiler, these have not generally been available to users of these reports such as policy-makers and programme managers in the country. Model questionnaires in DHS-V and DHS-VI also used a similar approach with a reproductive calendar. However, a few countries modified the questionnaire to add additional questions or prompts to seek to improve the capture of stillbirths. Most standard reports from DHS-V onwards contain an estimate of perinatal mortality. In view of ongoing data quality concerns about the information collected regarding non-live births using the reproductive calendar in previous phases, DHS-VII introduced a new table in sections 2–3 to record the details of all non-live births in the last 5 years including the month and year of the event and the length of gestation in months, and a prompt “*since January 2010* (YEAR VARIES DEPENDING ON YEAR OF INTERVIEW*) have you had any other pregnancies that did not result in a live birth*?” (Fig. 3 and Additional file 2). This is effectively a back-truncated history for non-live births.

Unlike previous phases that asked for only the month and year of birth, the DHS-VII model questionnaire has a modification in wording to include asking for the day of birth [17]. This change also led to the introduction of the Century Day Code, an algorithm used to standardise dates for DHS survey across all surveys. Previously, the DHS has used the Century Month Code [38].

### Data for stillbirths and neonatal deaths in DHS phases I–VII

**Fig. 4:**
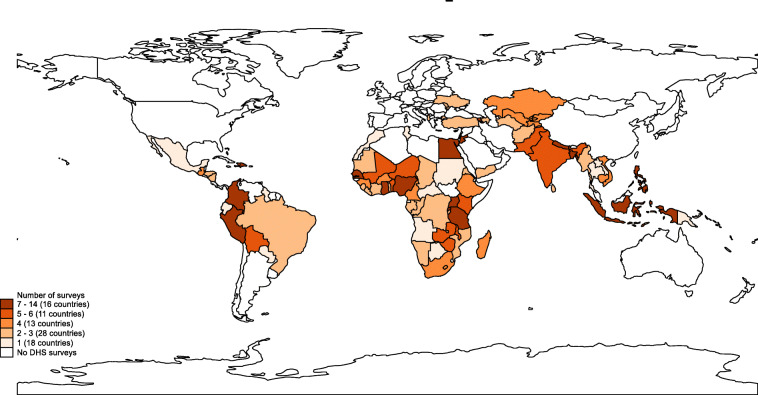
Demographic and Health Surveys (DHS) programme number of surveys in phases I–VII by country

**Fig. 5:**
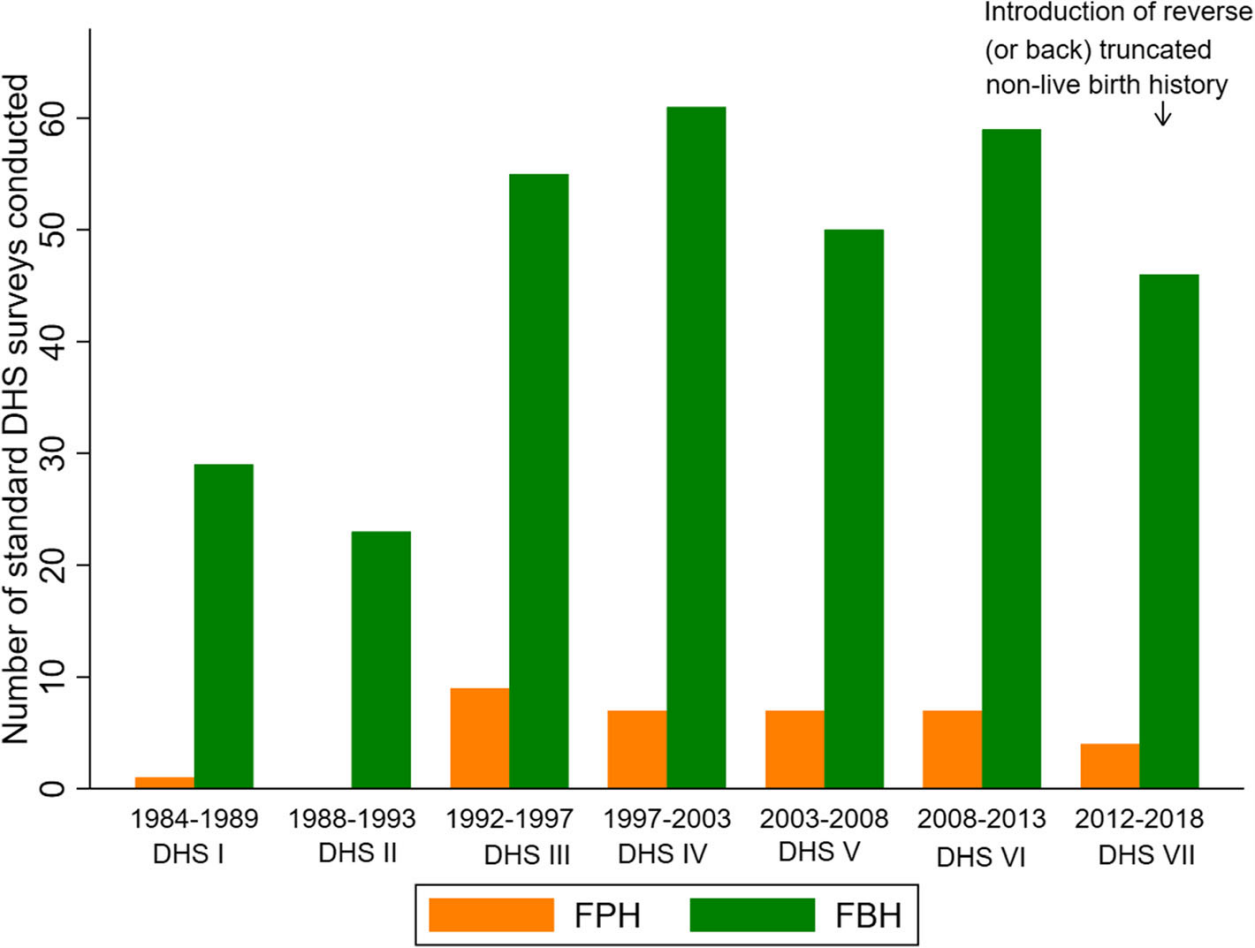
Demographic and Health Surveys phases I–VII (1985 to 2018) by type of maternity history

More than 350 surveys have been conducted in over 90 countries since 1984 (Fig. 4, Additional file 3 A and B) in the DHS programme. A FBH has been included in the standard model questionnaire and has been the predominant maternity history used in all phases of the programme so far (Figs. 5 and 3 and Additional files 4 and 5). Whilst country-specific adaptations can be undertaken, most countries have implemented the model questionnaire, although a report on DHS surveys in 27 countries in phase I found some changes in the question numbering and ordering [39].

**Fig. 6:**
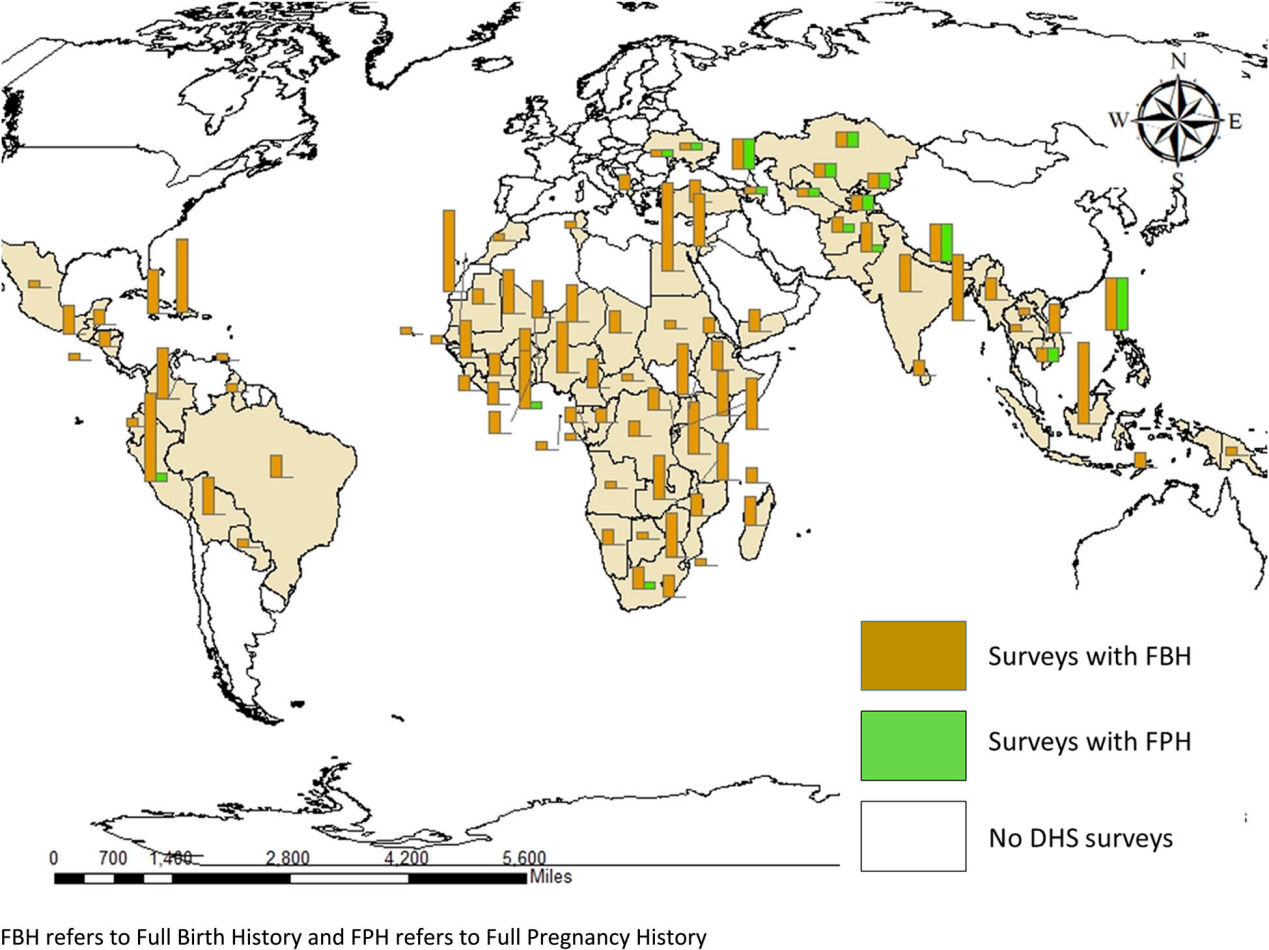
Location of Demographic and Health Surveys programme countries with FBH and FPH (phases I–VII)

An FPH module has been used in only 19% of countries that have conducted DHS. In 1986, Peru conducted the first DHS with an FPH module in an experiment to introduce questions around termination of pregnancies [40]. The other 16 countries started implementing an FPH from phase III (Additional file 4). A total of 35 and 323 FPH and FBH surveys, respectively, have been conducted since 1984 (see Fig. 6 and Additional files 4 and 5). Overall, some Asian and European countries use an FPH whilst only Ghana, South Africa and Peru from Africa and Latin America have (Fig. 6).

Initially, the DHS data collection was conducted using a paper-based system with data entry and processing done using the Census and Survey Processing System (CSPro). In the most recent phase of the DHS, both paper and computer-assisted personal interviewing (CAPI) were used. The DHS programme is planning to use the CSPro computer-assisted interviewing system for all future surveys [38, 41].

### Data quality for stillbirths and neonatal mortality for DHS phases I–VII

Data quality may vary by maternity history approach [42]. Changes to the model DHS questionnaire over time designed to improve stillbirth or neonatal death data quality include the changes in prompts to seek to reduce omission of live births who died from the FBH over phases III–V and including additional questions for non-live births in DHS-VII (Fig. 3).

**Fig. 7:**
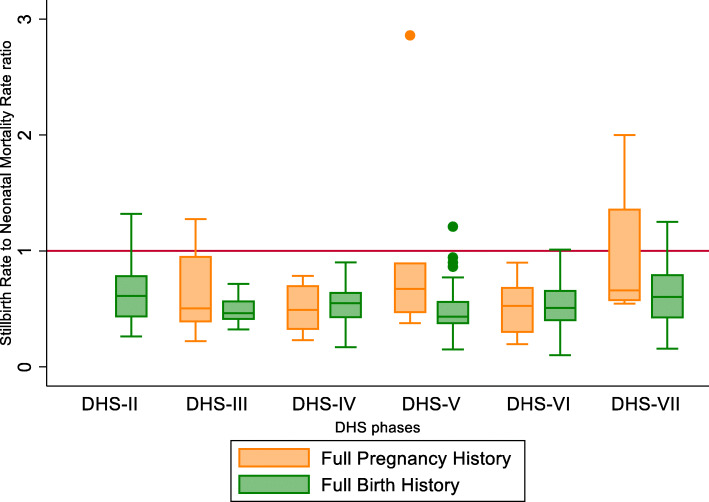
Distribution of stillbirth tate to neonatal mortality rate ratio by DHS module by phases II–VII

**Fig. 8A:**
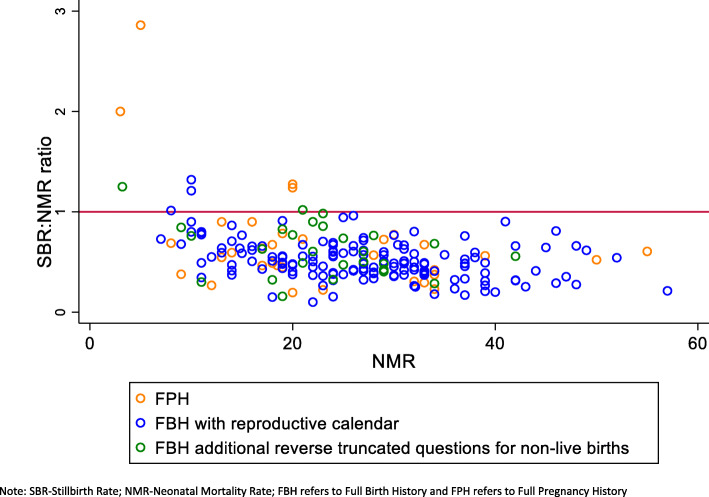
Comparison of DHS surveys (1990–2018) for SBR:NMR ratios a data quality marker by module

Overall, there was little variation in the SBR to NMR ratio in surveys with an FBH and reproductive calendar (RC) (DHS II-VI) and a slightly higher variation in surveys with an FBH with additional questions on pregnancy losses (DHS-VII), and all mean SBR:NMR ratios were below one (Table 1, Fig. 7). Only three surveys with an FBH had an SBR:NMR ratio > 1, 0.7% of those with a RC, and 5.9% of those with additional questions (Figs. 8a, b). The SBR to NMR ratio in the FPH was variable, implying that the FPH’s data quality was highly variable across DHS-III-VII, and all median SBR:NMR ratios were below one (Fig. 7). Four (11.7%) of the surveys had an SBR:NMR ratio >1 (Table 1 and Fig. 8a, b). We found no evidence of a trend in mean SBR:NMR in FBH or FPH across the ordered DHS (III–VII) (Table 1).

**Table 1:** DHS phases’ summary of neonatal mortality and stillbirth rates by maternity history type

DHS phase	Number of surveys (conducted)	Mean stillbirth rate (95%CI)^3^	Mean neonatal mortality rate (95%CI)^3^	Median SBR:NMR^4^	Median proportion of NND that are day 0-6 ENMR:NMR^5^
Full birth history					
I^1^	29				
II	23	14.08 (10.59–17.56)	24.38 (17.87–30.88)	0.61	0.69
III	54	13.89 (10.09–17.68)	28.13 (22.82–33.42)	0.47	0.69
IV	61	15.81 (13.51–18.11)	30.78 (27.27–34.29)	0.55	0.73
V	50	11.83 (10.21–13.45)	26.21 (22.84–29.57)^^2^	0.43	0.75
VI	59	11.90 (10.23–13.56)	24.63 (21.87–27.39)	0.51	0.78
VII	45	13.16 (10.81–15.53)^^1^	23.79 (20.70–26.89)^^3^	0.61	0.78
Full pregnancy history					
I^1^	1				
II	0				
III	9	15.10 (7.74 – 22.46)	24.94 (15.63–34.25)	0.51	0.74
IV	7	13.6 (8.00 – 19.20)	27.38 (19.09–35.66)	0.49	0.76
V	7	14.16 (7.96 – 20.36)	19.57 (9.70–29.44)^^2^	0.67	0.81
VI	7	12.10 (0.81 – 23.39)	24.66 (6.70–42.58)	0.53	0.77
VII	5	9.18 (2.51 – 15.84)^^1^	18.60 (0.51–36.69)^^3^	0.66	0.80

^1^Data collected, but neonatal mortality rate not disaggregated in the reports

^2^Stillbirth rates only available from surveys in countries with high contraceptive prevalence using model questionnaires 2A and 3A

^3^Proportion test comparing the mean stillbirth rate or mean neonatal mortality rate by survey module in each DHS phase: *p* = 0.016 in ^^1^; *p* = 0.026 in ^^3^; *p* = 0.020 in ^^3^

^4^Trend test comparing SBR:NMR for each maternity history type found “no trend” across the ordered DHS phases (full birth history: *p* = 0.949 and full pregnancy history: *p* = 0.317)

^5^DHS refers to the Demographic and Health Surveys; SB refers to stillbirths; NND refers to neonatal deaths; NMR refers to neonatal mortality rate; SBR refers to stillbirth rate; and ENMR refers to early neonatal mortality rate

*Survey SBR and NMR estimates extracted from DHS STATcompiler

Comparing the mean SBR between FBH and FPH by phase, we found evidence of a difference for estimates obtained in DHS-VII (*p* = 0.016). Similarly, evidence of a difference in the mean NMR estimates in DHS-V (*p* = 0.026) and DHS-VII (*p* = 0.020) was found (Table 1). For neonatal mortality, the ENMR:NMR ratio increased over time for both surveys using an FBH (*p* = 0.025) and an FPH (*p* = 0.070) (Table 1).

## Discussion

We reviewed the 35-year evolution of Demographic and Health Surveys’ model questionnaires since inception of the programme, covering over 90 countries and with more than 350 surveys, and synthesised implications for data on stillbirths and neonatal deaths. To the best of our knowledge, this is the first detailed overview of the measurement of stillbirths and neonatal deaths in DHS surveys over time examining the impact of different maternity histories modules and reproductive calendar approaches, including the resultant data quality. We focus on stillbirths and neonatal deaths because these are important indicators of maternal health, and universal healthcare access, utilisation and quality.

Most of the surveys used an FBH as provided in the DHS model questionnaire in each of the phases DHS-I to DHS-VII. Some changes have been made to the model questionnaire to improve data on stillbirths and neonatal deaths, most notably the inclusion of a reverse-truncated history for non-live births to capture information on stillbirths in the last 5 years and inclusion of a question on the day of birth in DHS-VII. We also found that the implementation of questions for collection of data on stillbirths in the woman’s questionnaire varies, with information captured as part of the main maternity history with an FPH approach, and in sub-section 2.3 for the reproductive calendar or additional non-live birth questions approaches used with an FBH [17].

Both FPH and FBH approaches seek to capture all child deaths including neonatal deaths which comprise around half of under-5 child deaths globally. Whilst FPH also captures information on stillbirths, the FBH has been supplemented with a reproductive calendar or additional questions on non-live births to capture such data in surveys since DHS-II. In practice, stillbirths, miscarriages and terminations of pregnancy are not fully captured, especially in FBH plus reproductive calendar or additional questions in the current DHS-VII [43]. Based on recent qualitative research, we hypothesise that this may be because the women do not report the event or timing, or do not perceive stillbirths as “valued”, or avoid reporting due to stigma in the community associating adverse outcomes with evil spirits [44]. Alternatively, the interviewers may omit or mis-record the event deliberately or unconsciously. Early neonatal deaths are live births, but may be misclassified as stillbirths [45] or also not reported, as these are also a source of stigma [46].

Our study found an increasing proportion of neonatal deaths that are in the early neonatal period. Whilst previous studies have reported that this proportion is stable across different levels of NMR, within survey data they also found that earlier surveys had on average lower proportions of deaths in the first week [25]. The changes found in the DHS model questionnaire regarding the capture of neonatal deaths were minor and are unlikely to have influenced the changes in the mortality estimates over time. Hence, observed changes may be due to true epidemiology variation, or changes in measurement such as reductions in the heaping on day 7, reductions in the omission of deaths due to increased societal recognition or changes in misclassification of stillbirths and neonatal deaths.

The high levels of variability in the SBR to NMR ratio observed across surveys with an FPH may reflect variation in data quality due to variations in the FPH tool and training between surveys. Whilst the standard FBH from the DHS model questionnaire was used in most FBH surveys, and as shown this varied little over time, no standard tool was available for FPH, and these varied both in questions asked and implementation across different countries.

A strength of our study is that it presents the first overall overview of the capture of stillbirths and neonatal deaths in DHS throughout, reviewing DHS model questionnaires, programme implementation and data on these outcomes throughout the lifetime of the programme from inception in 1984 to 2019. An important limitation of this study is that it only examines question similarities or differences in the questionnaires in terms of numbering, wording and interviewer instructions. In analysing data quality, only compiled data available via the STATcompiler were used; future studies could use survey microdata to further assess data quality aspects, such as heaping on day 7.

The most important limitation is the lack of robust measures for data quality for stillbirth and neonatal mortality rates. For stillbirths, we applied a simple test comparing the SBR to NMR ratio by survey modules, but this is simplistic and any potential variation with the level of NMR and SBR in LMICs is as yet poorly understood [7, 18]. SBR were lower than expected in almost all the surveys conducted by DHS phase, including both FBH and FPH, and this was consistent with findings from the EN-INDEPTH study [42].

A crucial research gap left unanswered by this study is how to better assess data quality for stillbirths. Unlike stillbirths, neonatal deaths have several data quality indicators developed, recommended and used to establish the quality of the data. To be able to improve the data over time requires measures that may be simple and generic, such as completeness, percentage of don’t knows, and if relevant (e.g. for continuous variables such as dates or birthweight), measures of heaping. However, for stillbirths, the main quality criterion SBR:NMR remains as yet poorly understood, and work is required to develop further methods to assess data quality in both survey and routine data. These measures would need to be validated against “gold standard” data with accurate measures of stillbirths, which require first trimester gestational age assessment or accurate birthweight and accurate assessment of signs of life at birth.

The DHS programme announced a major shift for DHS phase VIII by including for the first time an FPH in its model questionnaire [47]. The change followed findings from the EN-INDEPTH study, a randomised comparison of the DHS-VII model questionnaire’s FBH plus additional questions on recent non-live births to the most recent FPH implemented in Nepal. Evidence from that study suggested that FPH improved reporting of stillbirths, but had no effect for neonatal deaths [42]. However, whilst the FPH approach resulted in higher SBR estimates overall, this finding varied across sites underlining that changes to the questionnaire tools may be necessary but not sufficient, with other factors such as survey training and implementation likely playing an important role. In addition, the SBR estimates using an FPH were still lower than expected, assuming that, consistent with previous studies, an SBR:NMR of around 1 would be seen in these populations [18, 20, 21]. Implementation of the survey and also contextual barriers to reporting adverse pregnancy events such as stillbirths must also be considered and addressed [46].

## Conclusions

DHS remains the major data source for pregnancy outcomes worldwide. Although the DHS model questionnaire has evolved over the last three and a half decades, more robust evidence is required concerning optimal methods to obtain accurate data on stillbirths and neonatal deaths through household surveys.

The change in DHS-VIII from FBH to FPH is expected to improve the capture of data on pregnancy losses. However, whilst FPH may improve the capture of stillbirths, optimising data quality is also dependent on implementation including training and supervision of data collectors, and addressing contextual barriers to women reporting pregnancy losses. More research is required to develop and test standardised robust data quality measures for stillbirths and neonatal deaths for use in both survey and routine data. Investing in these will ensure that by the end of the Sustainable Development Goals era, countries have more data of higher quality to use for tracking their national targets and reducing these five million preventable neonatal deaths and stillbirths.

## Supplementary information

**Additional file 1:** Maternal history questions in the FBH and FPH modules (Section II)

**Additional file 2:** Comparing the DHS’s model FBH questionnaire across Phases I-VII

**Additional file 3: A**. Location of Demographic and Health Survey program countries and number of surveys, Phases I-IV. **B**. Location of Demographic and Health Survey program countries and number of surveys, Phases V-VII

**Additional file 4:** DHS surveys with Full Pregnancy History modules by DHS phase

**Additional file 5:** DHS surveys with Full Birth History modules by DHS phase

## Data Availability

This review used DHS data available from the DHS programme website on request (https://dhsprogram.com/What-We-Do/Survey-Types/DHS.cfm). All studies included in the review have been cited in the reference section.

## Abbreviations

DHS: Demographic and Health Surveys
EN-INDEPTH: Every Newborn - International Network for the Demographic Evaluation of Populations and their Health
FBH+: Full birth history (+ denotes additional questions on pregnancy losses)
FPH: Full pregnancy history
LMICs: Low- and middle-income countries
MICS: Multiple Indicator Cluster Survey
NMR(s): Neonatal mortality rate(s)
SB: Stillbirths
SBR(s): Stillbirth rate(s)
NND: Neonatal deaths

## References

[1] de Bernis L, Kinney MV, Stones W, ten Hoope-Bender P, Vivio D, Leisher SH, Bhutta ZA, Gülmezoglu M, Mathai M, Belizán JM (2016). Stillbirths: ending preventable deaths by 2030. Lancet.

[2] Lawn JE, Blencowe H, Oza S, You DZ, Lee ACC, Waiswa P, Lalli M, Bhutta Z, Barros AJD, Christian P (2014). Every newborn: progress, priorities, and potential beyond survival. Lancet.

[3] World Health Organization: Every newborn: an action plan to end preventable deaths. 2014. http://www.healthynewbornnetwork.org/hnn-content/uploads/Every_Newborn_Action_Plan-ENGLISH_updated_July2014.pdf. [Accessed November 2019].

[4] Cousens S, Blencowe H, Stanton C, Chou D, Ahmed S, Steinhardt L, Creanga AA, Tuncalp O, Balsara ZP, Gupta S (2011). National, regional, and worldwide estimates of stillbirth rates in 2009 with trends since 1995: a systematic analysis. Lancet.

[5] UN Inter-agency Group for Child Mortality Estimation: Levels and trends in child mortality. pp. 8; 2017:8.

[6] Heazell AEP, Siassakos D, Blencowe H, Burden C, Bhutta ZA, Cacciatore J, Dang N, Das J, Flenady V, Gold KJ (2016). Stillbirths: economic and psychosocial consequences. Lancet.

[7] Lawn JE, Blencowe H, Waiswa P, Amouzou A, Mathers C, Hogan D, Flenady V, Froen JF, Qureshi ZU, Calderwood C (2016). Stillbirths: rates, risk factors, and acceleration towards 2030. Lancet.

[8] Bhutta Z, Das J, Bahl R, Lawn JE, Salam RA, Paul VK, Sankar MJ, Blencowe H, Rizvi A, Chou V (2014). Can available interventions end preventable deaths in mothers, newborn babies, and stillbirths, and at what cost?. Lancet.

[9] Edouard L (1985). The epidemiology of perinatal mortality. World Health Statistics Quarterly.

[10] Walsh JA, Feifer CM, Measham AR, Gertler PJ: Maternal and perinatal health. 1993.

[11] AbouZahr C, De Savigny D, Mikkelsen L, Setel PW, Lozano R, Nichols E, Notzon F, Lopez AD (2015). Civil registration and vital statistics: progress in the data revolution for counting and accountability. Lancet.

[12] Cleland J (1996). Demographic data collection in less developed countries 1946–1996. Popul Stud.

[13] Boerma JT, Sommerfelt AE (1993). Demographic and Health Surveys (DHS): contributions and limitations. World Health Statistics Quarterly.

[14] Fabic MS, Choi Y, Bird S (2012). A systematic review of Demographic and Health Surveys: data availability and utilization for research. Bull World Health Organ.

[15] DHS Program: Demographic and Health Surveys. 2019. https://dhsprogram.com/What-We-Do/Survey-Types/DHS.cfm. [Accessed January 2020].

[16] Corsi DJ, Neuman M, Finlay JE, Subramanian SV (2012). Demographic and health surveys: a profile. Int J Epidemiol.

[17] Demographic and Health Surveys: DHS model questionnaires. 2018. https://dhsprogram.com/What-We-Do/Survey-Types/DHS-Questionnaires.cfm.[Accessed November 2019].

[18] Blencowe H, Cousens S, Jassir FB, Say L, Chou D, Mathers C, Hogan D, Shiekh S, Qureshi ZU, You D (2016). National, regional, and worldwide estimates of stillbirth rates in 2015, with trends from 2000: a systematic analysis. Lancet Glob Health.

[19] Bradley SEK, Winfrey W, Croft TN: Contraceptive use and perinatal mortality in the DHS: an assessment of the quality and consistency of calendars and histories. In DHS Methodological Reports, vol. 17. Rockville: ICF International; 2015.

[20] Butler N (1961). Perinatal mortality survey under the auspices of the National Birthday Trust Fund (preliminary communication). Organization and returns. Proc R Soc Med.

[21] World Health Organization: Neonatal and perinatal mortality for the year 2000: country, regional and global estimates. 2006. http://apps.who.int/iris/bitstream/handle/10665/43444/9241563206_eng.pdf;jsessionid=0712A8D434A36DF7FD37667960C32E89?sequence=1 [Accessed November 2019].

[22] Pullum T, Becker S (2014). Evidence of omission and displacement in DHS birth histories: methodological reports vol. 11.

[23] UN Inter-agency Group for Child Mortality Estimation: Levels and trends in child mortality. United Nations Inter-agency Group for Child Mortality Estimation (UN IGME); 2019. 10.1371/journal.pone.0101112 PMC4094389 25013954

[24] Oestergaard MZ, Inoue M, Yoshida S, Mahanani WR, Gore FM, Cousens S, Lawn JE, Mathers CD, Grp UNI-A, Epidemiology CH (2011). Neonatal mortality levels for 193 countries in 2009 with trends since 1990: a systematic analysis of progress, projections, and priorities. PLoS Med.

[25] Oza S, Cousens SN, Lawn JE (2014). Estimation of daily risk of neonatal death, including the day of birth, in 186 countries in 2013: a vital-registration and modelling-based study. Lancet Glob Health.

[26] World Health Organization: International classification of diseases for mortality and morbidity statistics. 2019. https://icd.who.int/browse11/l-m/en#/http://id.who.int/icd/entity/1947342847. [Accessed December 2019].

[27] Hill K: Indirect estimation of child mortality. Tools for demographic estimation 2013:148-164.

[28] Coale AJ, Brass W: The demography of tropical Africa. In Methods of analysis and estimation. Princeton University Press, Princeton, NJ; 1968: 88-139.

[29] Rajaratnam JK, Tran LN, Lopez AD, Murray CJL (2010). Measuring under-five mortality: validation of new low-cost methods. PLoS Med.

[30] Espeut D, Becker S (2015). The validity of birth and pregnancy histories in rural Bangladesh. J Health Popul Nutr.

[31] Becker S, Mahmud S: A validation study of backward and forward pregnancy histories in Matlab Bangladesh. International Statistical Institute; 1984.

[32] Thompson L, Ali MN, Casterline JB: Collecting demographic data in Bangladesh: evidence from tape-recorded interviews. In. International Statistical Institute; 1982.

[33] Goldman N, Moreno L, Westoff CF (1989). Peru experimental study: an evaluation of fertility and child health information.

[34] Westoff CF, Goldman N, Moreno L (1990). Dominican republic experimental study an evaluation of fertility and child health information.

[35] Goldman N, Moreno L, Westoff CF (1989). Collection of survey data on contraception - an evaluation of an experiment in Peru. Stud Fam Plan.

[36] Moreno L, Goldman N, Babakol O (1991). Use of a monthly calendar for collecting retrospective data on contraception: an evaluation of the experimental field studies of the Demographic and Health Surveys (DHS). Notas Poblacion.

[37] MacQuarrie KLD, Winfrey W, Meijer-Irons J, Morse A: Consistency of reporting of terminated pregnancies in DHS calendar. In DHS Methodological Reports No 25. pp. 6-7. Rockville, Maryland, USA: ICF; 2018:6-7.

[38] Croft T, Marshall AMJ, Allen CK (2018). Guide to DHS statistics.

[39] Landers A, McNiff M: Comparability of questionnaires. In DHS Methodological Reports, vol. 4. Calverton: Macro International Inc; 1994.

[40] Huntington D, Mensch B, Toubia N (1993). A new approach to eliciting information about induced-abortion. Stud Fam Plan.

[41] United States Census Bureau: Census and Survey Processing System (CSPro). 2019. https://www.census.gov/data/software/cspro.html. [Accessed December 2019].

[42] Akuze J, Blencowe H, Waiswa P, Baschieri A, Gordeev VS, Kwesiga D, Fisker AB, Thysen SM, Rodrigues A, Biks GA, et al: Randomised comparison of two household survey modules for measuring stillbirths and neonatal deaths in 69,176 pregnancies in five countries: the Every Newborn-INDEPTH study. Lancet Global Health 2020;8:E555–66. 10.1016/S2214-109X(20)30044-9 32199123

[43] Christou A, Alam A, Hofiani SMS, Rasooly MH, Mubasher A, Rashidi MK, Dibley MJ, Raynes-Greenow C (2019). How community and healthcare provider perceptions, practices and experiences influence reporting, disclosure and data collection on stillbirth: Findings of a qualitative study in Afghanistan. Soc Sci Med.

[44] Kiguli J, Namusoko S, Kerber K, Peterson S, Waiswa P (2015). Weeping in silence: community experiences of stillbirths in rural eastern Uganda. Glob Health Action.

[45] Liu L, Kalter HD, Chu Y, Kazmi N, Koffi AK, Amouzou A, Joos O, Munos M, Black RE (2016). Understanding misclassification between neonatal deaths and stillbirths: empirical evidence from Malawi. PLoS One.

[46] Kwesiga D, Tawiah C, Imam A, Kebede A, Nareeba T, Enuameh YA, Manu G, Beedle A, Fisker A, Waiswa P, et al.: Barriers and enablers to reporting of pregnancy and adverse pregnancy outcomes in population-based surveys: EN-INDEPTH multi-country study [in press]. BMC Population Health Metrics 2020. 10.1186/s12963-020-00228-x PMC7869448 33557858

[47] Demographic and Health Surveys: DHS8 Questionnaire Summary: revision process and new content. DHS; 2019.

