# Supplementary material for: Four decades of measuring stillbirths and neonatal deaths in Demographic and Health Surveys: historical review
Source: Popul Health Metr. 2021 Feb 8;19(Suppl 1):8. doi: 10.1186/s12963-020-00225-0 (PMC7869207; doi:10.1186/s12963-020-00225-0)
Supplement: Supplementary file 1 — Additional file 1: Maternal history questions in the FBH and FPH modules (Section II) [file 12963_2020_225_MOESM1_ESM.docx]

# **Additional file 1: Maternity history questions in the FBH and FPH modules**

The FBH and FPH questions are listed below using generic question numbers with “Q’s” and “P’s” representing FBH and FPH respectively.

Birth history

*Q211. “Now I would like to record the names of all your births, whether still alive or not, starting with the first one you had.”*

*Q212. “What name was given to your [first/next] baby?”*

*Q213. “Is [NAME] a boy or a girl?”*

*Q214. “Were any of these births twins?” (Note: each child in a multiple birth is listed separately)*

*Q215. “In what month and year was [NAME] born?”*

*Q216. “Is [NAME] still alive?”*

*Q217. “If alive: How old was [NAME] at his/her last birthday?”*

*Q218. “If alive: Is [NAME] living with you?”*

*Q219. If alive: interviewer records the household line number of the child*

*Q220. If dead: “How old was [NAME] when he/she died” (Note: see detail below)*

*Q221. “Were there any other live births between [NAME OF PREVIOUS BIRTH] and [NAME], including any children who died after birth?”*

Pregnancy History^^[[1]](#footnote-1)^^

*P211. "Now I would like to record all your pregnancies, whether born alive, born dead, or lost before full term, starting with the first one you had."*

*P212. "Pregnancy history number"*

*P212A. "Think back to your first/next pregnancy. Was that a single or multiple pregnancy?"*

*P212B. "Was the baby born alive, born dead, or lost before birth?"*

*P212C. "Did that baby cry, move, or breathe when it was born?"*

*P212D. "What name was given to the child?"*

*P213. "Is (NAME) a boy or a girl?"*

*P215. "On what day, month, and year was (NAME) born?"*

*P216. "Is (NAME) still alive?"*

*P217. "How old was (NAME) at (NAME)'s last birthday?"*

*P218. "Is (NAME) living with you?"*

*P219. "RECORD HOUSEHOLD LINE NUMBER OF CHILD. RECORD '00' IF CHILD NOT LISTED IN HOUSEHOLD."*

*P220. "How old was (NAME) when (he/she) died?"*

*IF '12 MONTHS' OR '1 YR', ASK: Did (NAME) have (his/her) first birthday?*

*THEN ASK: Exactly how many months old was (NAME) when (he/she) died?"*

*P220AA. "On what day, month, and year did (NAME) die?"*

*P220AB. "On what day, month, and year did this pregnancy end?"*

*P220AC. "How many months did this pregnancy last?"*

*P220AD. "Did you or someone else do something to end this pregnancy?"*

*P221. "Were there any other pregnancies between the previous pregnancy and this pregnancy?"*

1. For pregnancy history questionnaire, questions P216, P217, P218 and P219 are asked if born alive and still living, questions P P220 and P220AA are asked if born alive and now dead, and questions P220AB, P220AC and P220AD are asked if born dead or lost before birth [↑](#footnote-ref-1)
