# Supplementary material for: Four decades of measuring stillbirths and neonatal deaths in Demographic and Health Surveys: historical review
Source: Popul Health Metr. 2021 Feb 8;19(Suppl 1):8. doi: 10.1186/s12963-020-00225-0 (PMC7869207; doi:10.1186/s12963-020-00225-0)
Supplement: Supplementary file 2 — Additional file 2: Comparing the DHS’s model FBH questionnaire across Phases I-VII [file 12963_2020_225_MOESM2_ESM.docx]

# **Additional file 2: Comparing the DHS’s model FBH questionnaire across phases I-VII**

| **QUESTIONS IN DHS’S MODEL QUESTIONNAIRE (MATERNITY HISTORY)** | **DHS PHASES** | | | | | | | | | | | **Section** |
| --- | --- | --- | --- | --- | --- | --- | --- | --- | --- | --- | --- | --- |
|  | **I-A** | **I-B** | **II-A** | **II-B** | **III-A** | **III-B** | **IV-A** | **IV-B** | **V** | **VI** | **VII** |  |
| Now I would like to ask about all the births you have had during your life. Have you ever given birth? | 201 | 201 | 201 | 201 | 201 | 201 | 201 | 201 | 201 | 201 | 201 | 2.1 |
| Do you have any sons or daughters to whom you have given birth who are now living with you? | 202 | 202 | 202 | 202 | 202 | 202 | 202 | 202 | 202 | 202 | 202 | 2.1 |
| a) How many sons live with you?  b) And how many daughters live with you? | 203 | 203 | 203 | 203 | 203 | 203 | 203 | 203 | 203 | 203 | 203 | 2.1 |
| Do you have any sons or daughters to whom you have given birth who are alive but do not live with you? | 204 | 204 | 204 | 204 | 204 | 204 | 204 | 204 | 204 | 204 | 204 | 2.1 |
| a) How many sons are alive but do not live with you?  b) And how many daughters are alive but do not live with you? | 205 | 205 | 205 | 205 | 205 | 205 | 205 | 205 | 205 | 205 | 205 | 2.1 |
| Have you ever given birth to a boy or girl who was born alive but later died? | 206 | 206 | 206 | 206 | 206 | 206 | 206 | 206 | 206 | 206 | 206 | 2.1 |
| a) How many boys have died? b) And how many girls have died? | 207 | 207 | 207 | 207 | 207 | 207 | 207 | 207 | 207 | 207 | 207 | 2.1 |
| SUM ANSWERS TO 203, 205, AND 207, AND ENTER TOTAL. IF NONE, RECORD '00'. | 208 | 208 | 208 | 208 | 208 | 208 | 208 | 208 | 208 | 208 | 208 | 2.1 |
| CHECK 208: Just to make sure that I have this right: you have had in TOTAL _____ births during your life. Is that correct? | 209 | 209 | 209 | 209 | 209 | 209 | 209 | 209 | 209 | 209 | 209 | 2.1 |
| 210: CHECK 208:  ONE OR MORE BIRTHS  NO BIRTHS | 210 | 210 | 210 | 210 | 210 | 210 | 210 | 210 | 210 | 210 | 210 | 2.1 |
| Now I would like to record the names of all your births, whether still alive or not, starting with the first one you had. | 211 | 211 | 211 | 211 | 211 | 211 | 211 | 211 | 211 | 211 | 211 | 2.2 |
| What name was given to your (first/ next) baby? | 212 | 212 | 212 | 212 | 212 | 212 | 212 | 212 | 212 | 212 | 212 | 2.2 |
| Is (NAME) a boy or a girl? | 213 | 213 | 214 | 214 | 214 | 214 | 214 | 214 | 214 | 213 | 213 | 2.2 |
| Were any of these births twins? | ··· | ··· | 213 | 213 | 213 | 213 | 213 | 213 | 213 | 214 | 214 | 2.2 |
| On what day, month, and year was (NAME) born? | 214 | 214 | 215 | 215 | 215 | 215 | 215 | 215 | 215 | 215 | 215# | 2.2 |
| Is (NAME) still alive? | 215 | 215 | 216 | 216 | 216 | 216 | 216 | 216 | 216 | 216 | 216 | 2.2 |
| IF ALIVE: How old was (NAME) at (NAME)'s last birthday? | 217 | 217 | 217 | 217 | 217 | 217 | 217 | 217 | 217 | 217 | 217 | 2.2 |
| IF ALIVE: Is (NAME) living with you? | 218 | 218 | 218 | 218 | 218 | 218 | 218 | 218 | 218 | 218 | 218 | 2.2 |
| IF ALIVE: record household line number of child. Record '00' if child not listed in household. | ··· | ··· | 219 | 219 | ··· | 219 | 219 | 219 | 219 | 219 | 219 | 2.2 |
| IF DEAD: How old was (NAME) when (he/she) died? IF '12 MONTHS' OR '1 YR', ASK: Did (NAME) have (his/her) first birthday? THEN ASK: Exactly how many months old was (NAME) when (he/she) died? RECORD DAYS IF LESS THAN 1 MONTH; MONTHS IF LESS THAN TWO YEARS; OR YEARS. | 216 | 216 | 220 | 220 | 219 | 220 | 220 | 220 | 220 | 220 | 220++ | 2.2 |
| Were there any other live births between (NAME OF PREVIOUS BIRTH) and (NAME), including any children who died after birth? | ··· | ··· | ··· | ··· | 221 | ··· | 221 | 221 | 221 | 221 | 221 | 2.2 |
| Have you had any live births since the birth of (NAME OF LAST BIRTH)? | ··· | ··· | ··· | ··· | 223 | ··· | 222 | 222 | 222 | 222 | 222 | 2.3 |
| COMPARE 208 WITH NUMBER OF BIRTHS IN BIRTH HISTORY  1. Numbers are the same 2. Numbers are different | 219 | 219 | 221 | 221 | 224 | 221 | 223 | 223 | 223 | 223 | 223 | 2.3 |
| CHECK 215: ENTER THE NUMBER OF BIRTHS IN [YEAR] –[YEAR]  NUMBER OF BIRTHS   NONE | ··· | ··· | 222 | 222 | 225 | 222 | 224 | 224 | 224 | 224 | 224 | 2.3 |
| CALENDER: "FOR EACH BIRTH IN " & FIVE_YRS_BEFORE_SRVY & "-" & FW_YR & ", ENTER 'B' IN THE MONTH OF BIRTH IN THE CALENDAR. WRITE THE NAME OF THE CHILD TO THE LEFT OF THE 'B' CODE. " & " FOR EACH BIRTH, ASK THE NUMBER OF COMPLETED MONTHS THE PREGNANCY LASTED AND RECORD 'P' IN EACH OF THE PRECEDING MONTHS ACCORDING TO THE DURATION OF PREGNANCY. " & "(NOTE: THE NUMBER OF 'P's MUST BE ONE LESS THAN THE NUMBER OF MONTHS THAT THE PREGNANCY LASTED.)" | ··· | ··· | 223 |  | 226 |  | 225 | 225 | 225 | 225 | 225 | 2.3 |
| Are you pregnant now? | 220 | 220 | 225 | 223 | 227 | 223 | 226 | 226 | 226 | 226 | 226 | 2.3 |
| How many months pregnant are you? RECORD NUMBER OF COMPLETED MONTHS. CALENDAR:  ENTER 'P's IN THE CALENDAR, BEGINNING WITH THE MONTH OF INTERVIEW AND FOR THE TOTAL NUMBER OF COMPLETED MONTHS. | 222 | 222 | 226 | 224 | 228 | 234 | 227 | 227 | 227 | 227 | 227 | 2.3 |
| When you got pregnant, did you want to get pregnant at that time? | ··· | ··· | 227 | 225 | 229 | 225 | 228 | 228 | 228 | 228 | 228 | 2.3 |
| CHECK 208: TOTAL NUMBER OF BIRTHS  a) Did you want to have a baby later on or did you not want any more children? b) Did you want to have a baby later on or did you not want any children? | ··· | ··· | ··· | ··· | ··· | ··· | ··· | ··· | ··· | 229++ | 229++ | 2.3 |
| Have you ever had a pregnancy that miscarried, was aborted, or ended in a stillbirth? | ··· | ··· | 228 | ··· | 230 | ··· | 229 | 229 | 229 | 230 | 230 | 2.3 |
| When did the last such pregnancy end? MONTH AND YEAR | ··· | ··· | 229 | ··· | 231 | ··· | 230 | 230 | 230 | 231 | 231 | 2.3 |
| CHECK 231: LAST PREGNACY ENDED IN [YEAR]-[YEAR] (< 5 YEARS) LAST PREGNACY ENDED IN [YEAR] OR EARLIER (>5 YEARS) | ··· | ··· | 230 | ··· | 232 | ··· | 231 | 231 | 231 | 232 | 232 | 2.3 |
| In what month and year did the preceding such pregnancy end? | ··· | ··· | ··· | ··· | ··· | ··· | ··· | ··· | ··· | ··· | 233++ | 2.3 |
| How many months pregnant were you when that pregnancy ended? | ··· | ··· | 231 | ··· | 233# | ··· | 232# | 232# | 232# | 233 | 234 | 2.3 |
| Since January [YEAR] (<5 YEAR BEFORE SURVEY), have you had any other pregnancies that did not result in a live birth? | ··· | ··· | ··· | ··· | 235 | ··· | 233 | 233 | 233 | 234 | 235 | 2.3 |
| CALENDER: "FOR EACH PREGNANCY THAT DID NOT END IN A LIVE BIRTH IN "& FIVE_YRS_BEFORE_SRVY & "-" & FW_YR & " OR LATER, ENTER 'T' IN THE CALENDAR IN THE MONTH THAT THE PREGNANCY TERMINATED AND 'P' FOR THE REMAINING NUMBER OF COMPLETED MONTHS OF PREGNANCY." & " IF THERE ARE MORE THAN FOUR PREGNANCIES THAT DID NOT END IN A LIVE BIRTH, USE AN ADDITIONAL QUESTIONNAIRE STARTING ON THE SECOND LINE." | ··· | ··· | 233# | ··· | 235# | ··· | 234# | 234# | 234# | 235# | 236 | 2.3 |
| Did you have any miscarriages, abortions or stillbirths that ended before [YEAR] (5 YEARS BEFORE SURVEY)? | ··· | ··· | 232 | ··· | C | ··· | 235 | 235 | 235 | 236 | 237 | 2.3 |
| When did the last such pregnancy that terminated before [YEAR] ((<5 YEAR BEFORE SURVEY)) end? | ··· | ··· | ··· | ··· | C | ··· | 236 | 236 | 236 | 237 | 238 | 2.3 |
| When did your last menstrual period start? | ··· | ··· | 234 | 226 | 236 | 226 | 237 | 237 | 237 | 238 | 239 | 2.3 |
| From one menstrual period to the next, are there certain days when a woman is more likely to become pregnant? | ··· | ··· | 235# | 227# | 237# | 227# | 238# | 238# | 238# | 239# | 240 | 2.3 |
| Is this time just before her period begins, during her period, right after her period has ended, or halfway between two periods? | ··· | ··· | ··· | ··· | ··· | ··· | 239 | 239 | 239 | 240 | 241 | 2.3 |
| After the birth of a child, can a woman become pregnant before her menstrual period has returned? | ··· | ··· | ··· | ··· | ··· | ··· | ··· | ··· | ··· | ··· | 242++ | 2.3 |
| During which times of the monthly cycle does a woman have the greatest chance of becoming pregnant? | ··· | ··· | 236++ | 228++ | 239 | 228 | ··· | ··· | ··· | ··· | ··· | ··· |
| *# - Question wording and or instructions is different*  *++ - New question introduced*  *C - Calendar* | | | | | | | | | | | | |
