# Supplementary material for: Four decades of measuring stillbirths and neonatal deaths in Demographic and Health Surveys: historical review
Source: Popul Health Metr. 2021 Feb 8;19(Suppl 1):8. doi: 10.1186/s12963-020-00225-0 (PMC7869207; doi:10.1186/s12963-020-00225-0)
Supplement: Supplementary file 3 — Additional file 3: A. Location of Demographic and Health Survey program countries and number of surveys, Phases I-IV. B. Location of Demographic and Health Survey program countries and number of surveys, Phases V-VII [file 12963_2020_225_MOESM3_ESM.docx]

**Additional file 3A: Location of Demographic and Health Survey program countries and number of surveys, Phases I -IV***
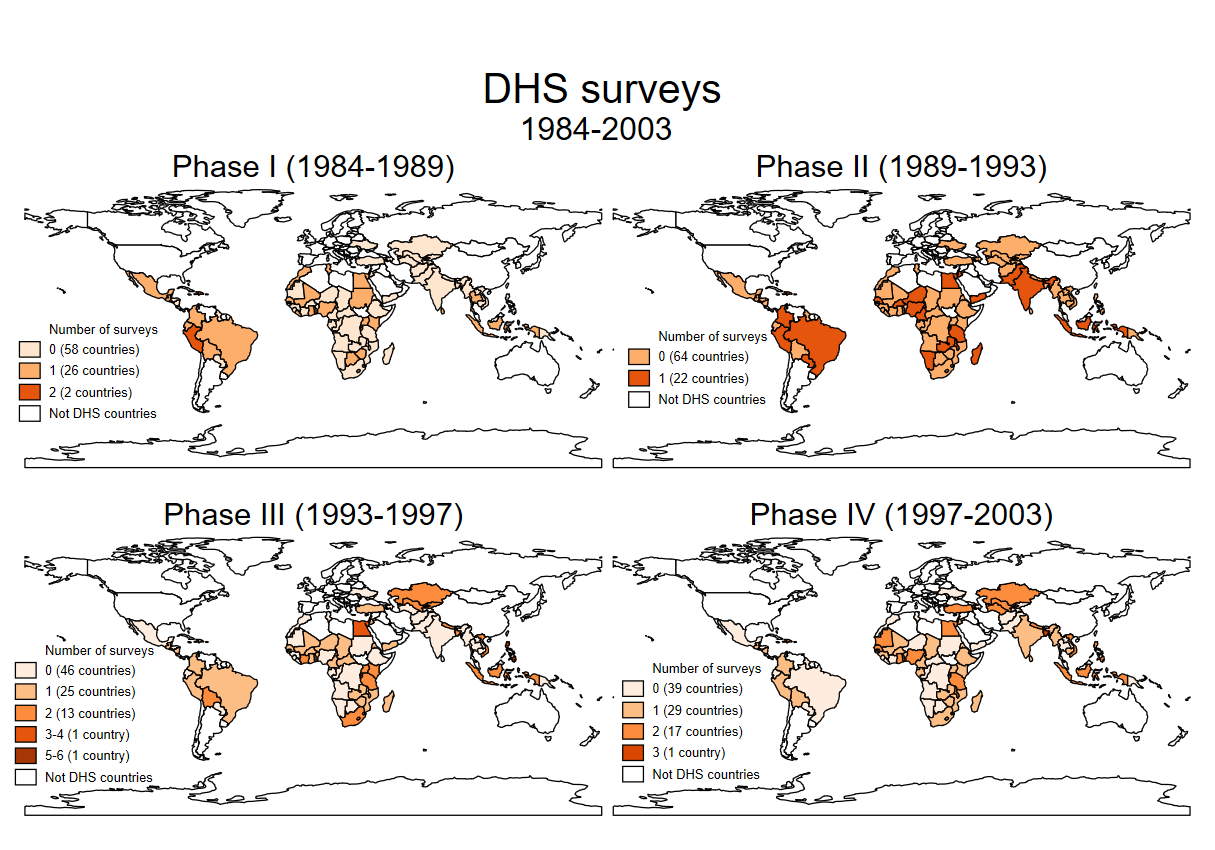
*

# **Additional file 3B: Location of Demographic and Health Survey program countries and number of surveys, Phases V- VII**

- **
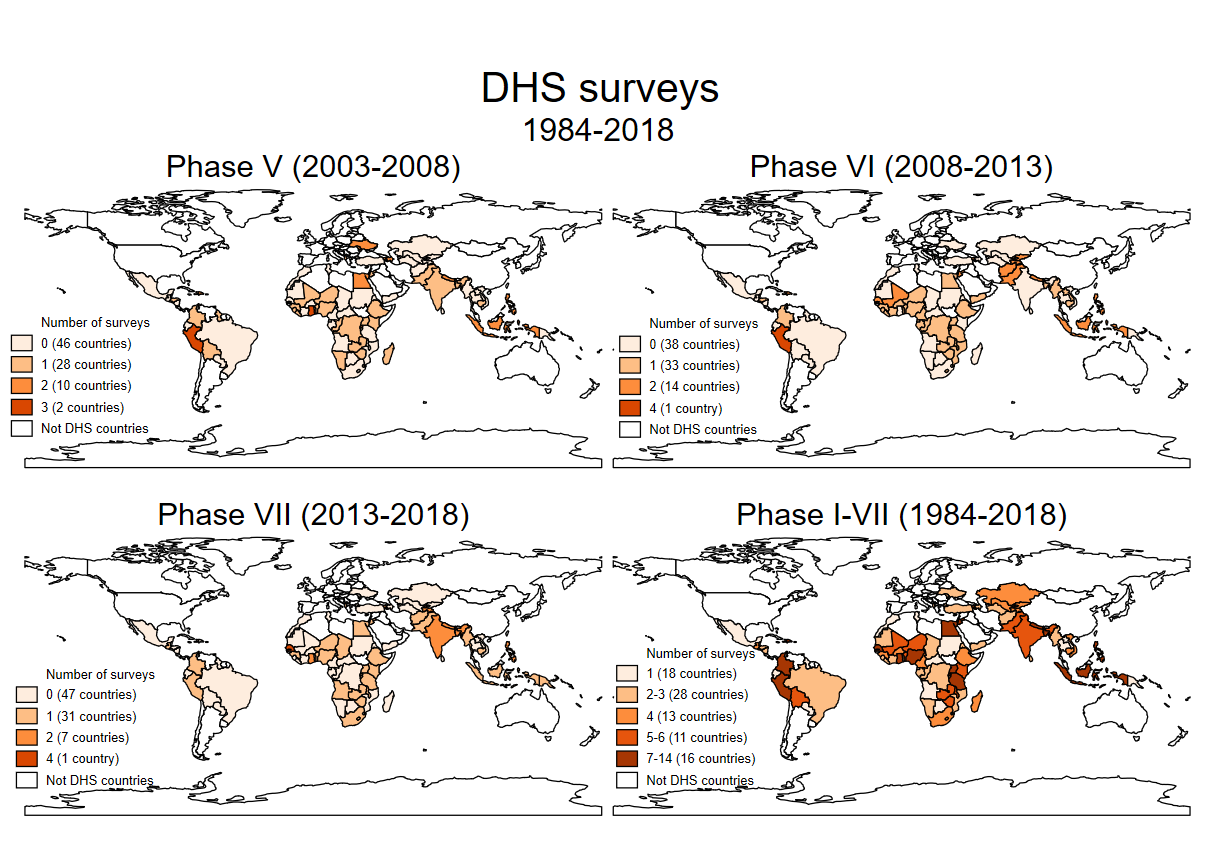
**
