# Supplementary material for: Four decades of measuring stillbirths and neonatal deaths in Demographic and Health Surveys: historical review
Source: Popul Health Metr. 2021 Feb 8;19(Suppl 1):8. doi: 10.1186/s12963-020-00225-0 (PMC7869207; doi:10.1186/s12963-020-00225-0)
Supplement: Supplementary file 4 — Additional file 4: DHS surveys with Full Pregnancy History modules by DHS phase [file 12963_2020_225_MOESM4_ESM.docx]

# **Additional file 4: DHS surveys with Full Pregnancy History modules by DHS phase**

| **country** | **1984-1989 DHS-I** | **1988-1993 DHS-II** | **1992-1997 DHS-III** | **1997-2003 DHS-IV** | **2NONO3-2008 DHS-V** | **2008-2013 DHS-VI** | **2013-2018 DHS-VII** | **Total** |
| --- | --- | --- | --- | --- | --- | --- | --- | --- |
| Afghanistan | NO | NO | NO | NO | NO | YES | NO | **1** |
| Armenia | NO | NO | NO | YES* | YES* | YES* | YES | **4** |
| Azerbaijan | NO | NO | NO | NO | YES* | NO | NO | **1** |
| Ghana | NO | NO | NO | NO | YES | NO | NO | **1** |
| Kazakhstan | NO | NO | YES | YES* | NO | NO | NO | **2** |
| Kyrgyz Republic | NO | NO | YES | NO | NO | YES | NO | **2** |
| Moldova | NO | NO | NO | NO | YES* | NO | NO | **1** |
| Nepal | NO | NO | YES | YES | YES | YES | YES | **5** |
| Pakistan | NO | NO | NO | NO | NO | YES | YES | **2** |
| Peru | YES | NO | NO | NO | NO | NO | NO | **1** |
| Philippines | NO | NO | YES (3) | YES | YES | YES | YES | **7** |
| South Africa | NO | NO | YES | NO | NO | NO | NO | **1** |
| Tajikistan | NO | NO | NO | NO | NO | YES | YES | **2** |
| Turkmenistan | NO | NO | NO | YES | NO | NO | NO | **1** |
| Ukraine | NO | NO | NO | NO | YES* | NO | NO | **1** |
| Uzbekistan | NO | NO | YES | YES | NO | NO | NO | **2** |
| Vietnam | NO | NO | YES* | YES* | NO | NO | NO | **2** |
| **Total** | **1** | NO | **9** | **7** | **7** | **7** | **5** | **36** |

*Surveys that used a backward approach
