# Supplementary material for: Four decades of measuring stillbirths and neonatal deaths in Demographic and Health Surveys: historical review
Source: Popul Health Metr. 2021 Feb 8;19(Suppl 1):8. doi: 10.1186/s12963-020-00225-0 (PMC7869207; doi:10.1186/s12963-020-00225-0)
Supplement: Supplementary file 5 — Additional file 5: DHS surveys with Full Birth History modules by DHS phase [file 12963_2020_225_MOESM5_ESM.docx]

# **Additional file 5: DHS surveys with Full Birth History modules by DHS phase**

|  | **PHASES** | | | | | | |  |
| --- | --- | --- | --- | --- | --- | --- | --- | --- |
| **country** | **1984-1989 DHS-I** | **1988-1993 DHS-II** | **1992-1997 DHS-III** | **1997-2003 DHS-IV** | **2003-2008 DHS-V** | **2008-2013 DHS-VI** | **2013-2018 DHS-VII** | **Total** |
| Afghanistan | NO | NO | NO | NO | NO | YES | YES | **2** |
| Albania | NO | NO | NO | NO | YES | NO | YES | **2** |
| Angola | NO | NO | NO | NO | NO | NO | YES | **1** |
| Armenia | NO | NO | NO | YES | YES | YES | YES | **4** |
| Azerbaijan | NO | NO | NO | NO | YES | NO | NO | **1** |
| Bangladesh | NO | NO | YES (2) | YES (3) | YES | YES | YES (2) | **9** |
| Benin | NO | NO | YES | YES | YES | YES | YES | **5** |
| Bolivia | YES | NO | YES (2) | YES | YES | NO | NO | **5** |
| Botswana | YES | NO | NO | NO | NO | NO | NO | **1** |
| Brazil | YES | YES | YES | NO | NO | NO | NO | **3** |
| Burkina Faso | NO | YES | YES | YES | NO | YES | NO | **4** |
| Burundi | YES | NO | NO | NO | NO | YES | YES | **3** |
| Cambodia | NO | NO | NO | YES | YES | YES | YES | **4** |
| Cameroon | NO | YES | YES | YES | NO | YES | NO | **4** |
| Cape Verde | NO | NO | NO | YES | NO | NO | NO | **1** |
| Central African Republic | NO | NO | YES | NO | NO | NO | NO | **1** |
| Chad | NO | NO | YES | YES | NO | NO | YES | **3** |
| Colombia | YES | YES | YES | YES | YES | YES | YES | **7** |
| Comoros | NO | NO | YES | NO | NO | YES | NO | **2** |
| Congo | NO | NO | NO | NO | YES | YES | NO | **2** |
| Congo Democratic Republic | NO | NO | NO | NO | YES | YES | NO | **2** |
| Cote d'Ivoire | NO | NO | YES (2) | NO | NO | YES | NO | **3** |
| Dominican Republic | YES (2) | YES | YES | YES (2) | YES (2) | YES (2) | NO | **10** |
| Ecuador | YES | NO | NO | NO | NO | NO | NO | **1** |
| Egypt | YES | YES | YES (4) | YES (2) | YES (2) | YES | YES | **12** |
| El Salvador | YES | NO | NO | NO | NO | NO | NO | **1** |
| Equatorial Guinea | NO | NO | NO | NO | NO | YES | NO | **1** |
| Eritrea | NO | NO | YES | YES | NO | NO | NO | **2** |
| Ethiopia | NO | NO | NO | YES | YES | YES | YES | **4** |
| Gabon | NO | NO | NO | YES | NO | YES | NO | **2** |
| Gambia | NO | NO | NO | NO | NO | YES | NO | **1** |
| Ghana | YES | NO | YES | YES (2) | YES (2) | NO | YES (2) | **8** |
| Guatemala | YES | NO | YES | YES | NO | NO | YES | **4** |
| Guinea | NO | NO | NO | YES | YES | YES | YES | **4** |
| Guyana | NO | NO | NO | NO | YES | NO | NO | **1** |
| Haiti | NO | NO | YES | YES | YES | YES (2) | YES | **6** |
| Honduras | NO | NO | NO | NO | YES | YES | NO | **2** |
| India | NO | YES | NO | YES | YES | NO | YES (2) | **5** |
| Indonesia | YES | YES | YES (2) | YES (2) | YES (2) | YES (2) | YES | **11** |
| Jordan | NO | YES | YES | YES | YES | YES (2) | YES | **7** |
| Kazakhstan | NO | NO | YES | YES | NO | NO | NO | **2** |
| Kenya | YES | NO | YES (2) | YES | YES | NO | YES | **6** |
| Kyrgyz Republic | NO | NO | YES | NO | NO | YES | NO | **2** |
| Lao People's Democratic Republic | NO | NO | NO | NO | NO | YES | NO | **1** |
| Lesotho | NO | NO | NO | YES | NO | YES | YES | **3** |
| Liberia | YES | NO | NO | NO | YES | YES | NO | **3** |
| Madagascar | NO | YES | YES | YES | YES | NO | NO | **4** |
| Malawi | NO | YES | NO | YES (2) | NO | YES | YES | **5** |
| Maldives | NO | NO | NO | NO | YES | NO | YES | **2** |
| Mali | YES | NO | YES | YES | YES | YES (2) | NO | **6** |
| Mauritania | NO | NO | NO | YES (2) | NO | NO | NO | **2** |
| Mexico | YES | NO | NO | NO | NO | NO | NO | **1** |
| Moldova | NO | NO | NO | NO | YES | NO | NO | **1** |
| Morocco | YES | YES | YES | YES | NO | NO | NO | **4** |
| Mozambique | NO | NO | YES | YES | NO | YES | NO | **3** |
| Myanmar | NO | NO | NO | YES | NO | YES | YES | **3** |
| Namibia | NO | YES | NO | NO | YES | NO | NO | **2** |
| Nepal | NO | NO | YES | YES | YES | YES | YES | **5** |
| Nicaragua | NO | NO | YES | YES | NO | NO | NO | **2** |
| Niger | NO | YES | YES | NO | YES | YES | YES | **5** |
| Nigeria | YES | YES | NO | YES (2) | YES | YES | YES | **7** |
| Pakistan | NO | YES | NO | NO | YES | YES | YES | **4** |
| Papua New Guinea | NO | NO | NO | NO | NO | NO | YES | **1** |
| Paraguay | NO | YES | NO | NO | NO | NO | NO | **1** |
| Peru | YES | YES | YES | YES | YES (3) | YES (4) | YES | **12** |
| Philippines | NO | NO | YES (3) | YES | YES | YES | YES | **7** |
| Rwanda | NO | YES | NO | YES | YES (2) | YES (2) | YES | **7** |
| Samoa | NO | NO | NO | NO | YES | NO | NO | **1** |
| Sao Tome and Principe | NO | NO | NO | NO | YES | NO | NO | **1** |
| Senegal | YES | YES | YES | YES (2) | NO | YES (2) | YES (4) | **11** |
| Sierra Leone | NO | NO | NO | NO | YES | YES | NO | **2** |
| South Africa | NO | NO | YES | YES | NO | NO | YES | **3** |
| Sri Lanka | YES | NO | NO | YES | NO | NO | NO | **2** |
| Sudan | YES | NO | NO | NO | NO | NO | NO | **1** |
| Swaziland | NO | NO | NO | NO | YES | NO | NO | **1** |
| Tajikistan | NO | NO | NO | NO | NO | YES | YES | **2** |
| Tanzania | NO | YES | YES (2) | YES (2) | NO | YES | YES | **7** |
| Thailand | YES | NO | NO | NO | NO | NO | NO | **1** |
| Timor-Leste | NO | NO | NO | NO | NO | YES | YES | **2** |
| Togo | YES | NO | YES | NO | NO | YES | NO | **3** |
| Trinidad and Tobago | YES | NO | NO | NO | NO | NO | NO | **1** |
| Tunisia | YES | NO | NO | NO | NO | NO | NO | **1** |
| Turkey | NO | NO | YES | YES (2) | NO | NO | NO | **3** |
| Turkmenistan | NO | NO | NO | YES | NO | NO | NO | **1** |
| Uganda | YES | NO | YES (2) | YES | YES | YES | YES | **7** |
| Ukraine | NO | NO | NO | NO | YES | NO | NO | **1** |
| Uzbekistan | NO | NO | YES | YES | NO | NO | NO | **2** |
| Vietnam | NO | NO | YES | YES | NO | NO | NO | **2** |
| Yemen | NO | YES | YES | NO | NO | YES | NO | **3** |
| Zambia | NO | YES | YES | YES | YES | YES | YES | **6** |
| Zimbabwe | YES | NO | YES | YES | YES | YES | YES | **6** |
| **Total** | **29** | **23** | **54** | **61** | **50** | **59** | **46** | **322** |
